# Papulonecrotic lesions and fever in an adult patient

**DOI:** 10.1016/j.jdcr.2024.08.041

**Published:** 2024-09-19

**Authors:** Daniel R. Antohi, Michelle Toker, Suraj Muddasani, Michael Occidental, Bijal Amin, Breanne Mordorski, Benedict Wu

**Affiliations:** a Albert Einstein College of Medicine, Bronx, New York; b Division of Dermatology, Department of Medicine, Montefiore Medical Center, Bronx, New York; c Department of Pathology, Montefiore Medical Center, Bronx, New York

**Keywords:** Mucha-Habermann disease, mycophenolate mofetil, papulosquamous dermatosis, pityriasis lichenoides et varioliformis acuta (PLEVA)

## Presentation

**Fig 1:**
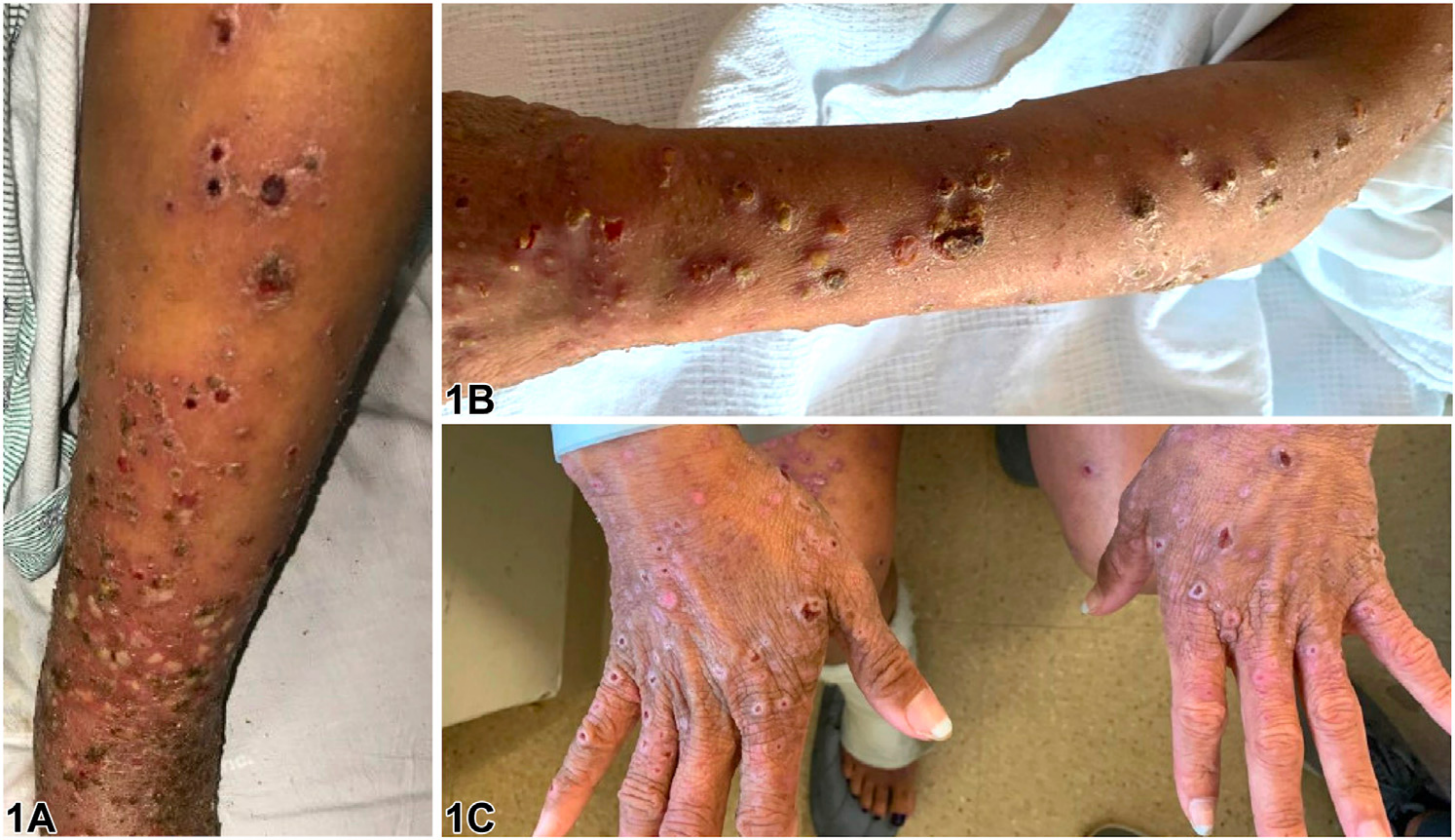
Upper extremities. **A,** Left arm at the time of presentation. **B,** Bilateral hands at presentation. **C,** Left leg at the time of presentation.

**Fig 2:**
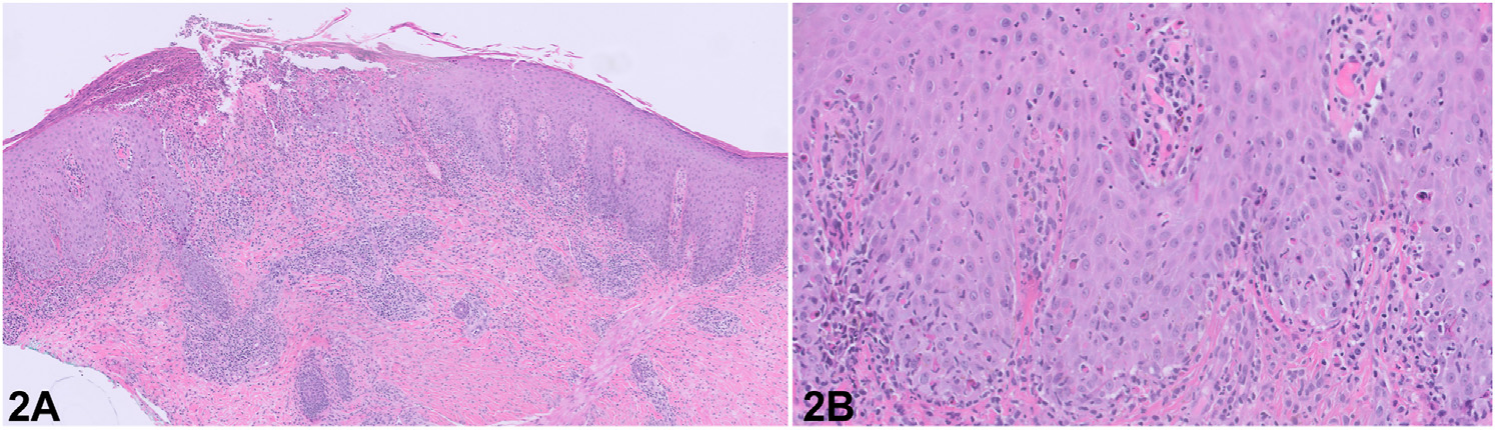
Histopathology. **A,** H&E, 5×. Low-power view shows an eroded epidermis with adjacent psoriasiform epidermal hyperplasia and lichenoid inflammation. **B,** H&E, 20×. There are numerous necrotic keratinocytes within the epidermis.

A 40-year-old woman with untreated eczema presented with a fever and a painful eruption. Physical examination revealed numerous crusted papulonecrotic erosions and round plaques on the trunk and extremities (Fig 1, *A-C*). Laboratory evaluation revealed negative antinuclear and extractable nuclear antigen autoantibodies, syphilis, human immunodeficiency virus, herpes simplex virus, varicella-zoster virus, and Epstein-Barr virus tests. The complete blood count and liver tests were unremarkable. A punch biopsy revealed an eroded epidermis with numerous necrotic keratinocytes and adjacent psoriasiform epidermal hyperplasia with a lichenoid and perivascular inflammatory cell infiltrate consisting of lymphocytes, plasma cells, and numerous neutrophils (Fig 2, *A* and *B*).

**Question 1: What is the most likely diagnosis based on the clinical presentation, laboratory studies, and histopathology?**

A. Lues maligna
B. Sweet syndrome
C. Bullous vasculitis
D. Mucha-Habermann disease
E. Guttate psoriasis

**Answers:**

A. Lues maligna – Incorrect. The negative syphilis IgM/IgG test militates against lues maligna, a severe variant of secondary syphilis.
B. Sweet syndrome – Incorrect. Sweet syndrome would show dense neutrophilic infiltrate on histopathology with papillary dermal edema and absence of lichenoid dermatitis with perivascular lymphocytes.
C. Bullous vasculitis – Incorrect. The histopathology failed to reveal features compatible with leukocytoclastic vasculitis.
D. Mucha-Habermann disease – Correct. Mucha-Habermann disease (MHD) is an exuberant and severe febrile form of pityriasis lichenoides et varioliformis acuta. MHD is characterized by parakeratosis, interface dermatitis with mainly lymphocytes and neutrophils, and extravasated erythrocytes on histopathology.^1^
E. Guttate psoriasis – Incorrect. Although psoriasiform hyperplasia can be seen in guttate psoriasis, a robust lichenoid inflammatory cell infiltrate with papulonecrotic lesions goes against guttate psoriasis.

**Question 2: Which of the following is the most appropriate initial treatment in this clinical setting?**

A. Phototherapy
B. Oral prednisone
C. Topical triamcinolone
D. Doxycycline
E. Methotrexate

**Answers:**

A. Phototherapy – Incorrect. The severe presentation with systemic symptoms would make phototherapy inappropriate.
B. Oral prednisone – Correct. Data regarding MHD treatment is limited, although commonly accepted first-line treatments include corticosteroids, systemic antibiotics, and phototherapy.^2^ Oral prednisone is appropriate, given its rapid onset of action.
C. Topical triamcinolone – Incorrect. For the widespread and ulcerative lesions, recommending topical therapies alone would not be appropriate.
D. Doxycycline – Incorrect. Although doxycycline is anti-inflammatory agent, the delayed onset of action would not be appropriate in this case.
E. Methotrexate – Incorrect. Similar to doxycycline, methotrexate would take longer to go into full effect and potentially lead to increased morbidity.

**Question 3: The decision was made to add on the steroid-sparing agent, mycophenolate mofetil (MMF); which of the following labs must be checked in this patient before starting MMF?**

A. Peripheral blood flow cytometry
B. Liver tests
C. Urinalysis
D. Complete blood count
Ε. β-human chorionic gonadotropin

**Answers:**

A. Peripheral blood flow cytometry – Incorrect. Peripheral blood flow cytometry would be checked if there is a concern for systemic lymphoma. MMF has been associated with an increased risk of lymphoma and skin malignancies, but this is irrelevant to this case.
B. Liver tests – Incorrect. MMF may cause minor, temporary elevations in liver enzymes but does not commonly exert liver toxicity in immunocompetent patients. However, MMF may have hepatotoxic effects on patients with a history of solid organ transplants.
C. Urinalysis – Incorrect. MMF can cause urinary symptoms such as urgency, frequency, and dysuria; MMF does not cause nephrotoxicity.^3^
D. Complete blood count – Incorrect. MMF can cause dose-dependent, reversible neutropenia, anemia, thrombocytopenia, and, on rare occasions, agranulocytosis.^4^
Ε. β-human chorionic gonadotropin – Correct. MMF is a teratogen and is associated with first-trimester loss, facial abnormalities, and anomalies of the distal limbs and other visceral organs.^5^

## Conflicts of interest

None disclosed.
